# Identification of MMP8, DDX24, RNASE2, and EMB as a novel diagnostic gene panel for sepsis: a transcriptome-based modeling study

**DOI:** 10.3389/fimmu.2026.1911134

**Published:** 2026-09-07

**Authors:** Zhiwen Gong, Xinyi Liu, Xiaoyu Xiang, Tingting Li, Zhongxue Feng, Jing Yang, Lietao Wang, Lijun Wang, Wei Zhang

**Affiliations:** 1 Institute of Critical Care Medicine, West China Hospital Sichuan University, Chengdu, China; 2 Department of Critical Care Medicine, Sichuan Clinical Research Center for Cancer, Sichuan Cancer Hospital and Institute, Sichuan Cancer Center, University of Electronic Science and Technology of China, Chengdu, China; 3 Department of Critical Care Medicine, State Key Laboratory of Biotherapy and Cancer Center, West China Hospital, Sichuan University and Collaborative Innovation Center of Biotherapy, Chengdu, Sichuan, China

**Keywords:** sepsis, early diagnosis, biomarker, diagnostic model, machine learning, immune

## Abstract

**Objective:**

Sepsis is a life-threatening condition with high mortality and complex pathology. Early diagnosis is critical but remains challenging due to a lack of effective biomarkers. This study aims to identify specific diagnostic markers to distinguish sepsis from non-sepsis, and to develop a robust diagnostic model.

**Methods:**

PBMC transcriptome data from our cohort (24 healthy controls, 29 common infections, 51 sepsis patients) were analyzed to identify genes with expression levels increasing or decreasing with infection severity. Low-expression genes were excluded, and candidate markers were evaluated using multiple GEO datasets. Top-performing genes were selected to build a LASSO regression-based diagnostic model. Model performance was assessed by AUC, BSS, ROC and calibration curves, nomogram, DCA, and CIC curves. Functional analyses (GO, KEGG, immune infiltration, GSEA, PPI network) were performed to explore underlying immune mechanisms.

**Results:**

A total of 114 genes showing expression changes with infection severity were identified. Four genes—MMP8, DDX24, RNASE2, and EMB—were selected based on diagnostic performance. The resulting model performed well in both our cohort and public datasets (AUC: 0.811–1; BSS: -0.464 – 0.964). In the GSE69686 dataset, the model also showed predictive value for neonatal and pediatric sepsis (AUC: 0.666–0.852; BSS: -1.34 – -0.293). Immune infiltration and GSEA revealed enrichment of these genes in neutrophils, monocytes, and T cells, reflecting key immune features of sepsis.

**Conclusion:**

The four identified genes—MMP8, DDX24, RNASE2, and EMB—collectively form a diagnostic model that effectively distinguishes sepsis patients from those with non-sepsis individuals.

## Introduction

Sepsis is a life-threatening clinical syndrome resulting from a dysregulated host response to infection (1, 2). The global burden of the condition is substantial, with an estimated 31.5 million cases and 5.3 million deaths annually (3). In China, sepsis affects approximately one in five patients in intensive care units (ICUs) and carries a 90-day mortality rate of 35.5% (4). Delays in antibiotic administration further worsen outcomes, as each hour of delay is associated with a significant increase in mortality (5, 6). Consequently, there is broad consensus that early diagnosis and prompt intervention are essential for improving prognosis.

According to the Sepsis-3.0 definition, the diagnostic focus has shifted from systemic inflammatory response syndrome (SIRS) to infection-induced organ dysfunction (7–9). In current clinical practice, infection is typically identified by measuring inflammatory markers such as procalcitonin (PCT) and C-reactive protein (CRP) (10), or by blood culture (11). Organ dysfunction is most often assessed using the Sequential Organ Failure Assessment (SOFA) or quick SOFA (qSOFA) score, with rapid bedside tools such as the National Early Warning Score (NEWS), NEWS2, and Modified Early Warning System (MEWS) also widely used (2, 12). However, these approaches have significant limitations for early diagnosis. First, scoring systems for organ dysfunction rely on biochemical and blood-gas parameters (13). By the time organ dysfunction is confirmed, the window for early intervention has already passed. Second, although blood culture is highly specific, it detects only 30-40% of cases and results typically take more than 48 hours (14, 15), which is too slow for timely decision-making. Finally, despite their utility in guiding antibiotic discontinuation (16), PCT and CRP lack the necessary sensitivity and specificity for dependable sepsis diagnosis (17–20). Thus, developing rapid, accurate, point-of-care biomarkers for early sepsis diagnosis remains a critical unmet need.

To address the issue of insufficient efficacy of current early diagnostic markers for sepsis, numerous studies have focused on identifying better candidates. Key nodes and related molecules involved in the immune response during sepsis are investigated and considered to have potential for early sepsis diagnosis. A recent study collected plasma samples from patients with acute infection and sepsis and screened four markers (Pentraxin-3, MyD88, GLP-1, and PD-L1) as potential tools for sepsis diagnosis and risk assessment (21). These four genes play important roles in regulating innate immune responses and immunosuppression, underscoring the value of investigating immune-related genes for biomarker discovery. Various analytical approaches have been applied to screen for biomarkers with diagnostic efficacy for sepsis, including transcriptome sequencing of peripheral blood immune cells, machine learning, and patient stratification based on immune status (22, 23). Nevertheless, challenges remain, such as small sample sizes, the “black-box” nature of machine learning algorithms, and the complex, heterogeneous course of sepsis. Consequently, further in-depth research is still needed to identify reliable early diagnostic markers for sepsis.

Our research team prospectively collected peripheral blood specimens from septic patients, patients with common infections, and healthy controls, and performed transcriptome sequencing of peripheral blood mononuclear cells (PBMCs). In this study, we will conduct an in-depth analysis of these sequencing data to reflect the severity of disease progression in septic patients based on infection severity, screening for differentially expressed genes (DEGs) that exhibit persistent changes with increasing infection severity. Furthermore, we will evaluate the diagnostic potential of these differentially expressed genes for sepsis across multiple public datasets, select those with the highest diagnostic potential to construct a sepsis diagnostic model, and systematically validate the model’s diagnostic performance, predictive accuracy, and clinical net benefit using several independent datasets. Based on the differential expression of these potentially diagnostic markers across groups, we will further perform immune infiltration analysis and gene set enrichment analysis to explore the potential mechanisms by which these genes function in the immunopathological processes of sepsis. This study aims to provide novel molecular markers and diagnostic models for the early identification and severity assessment of sepsis, laying the foundation for subsequent mechanistic investigations.

## Results

### Screening of differentially expressed genes in sepsis

**Figure 1:**
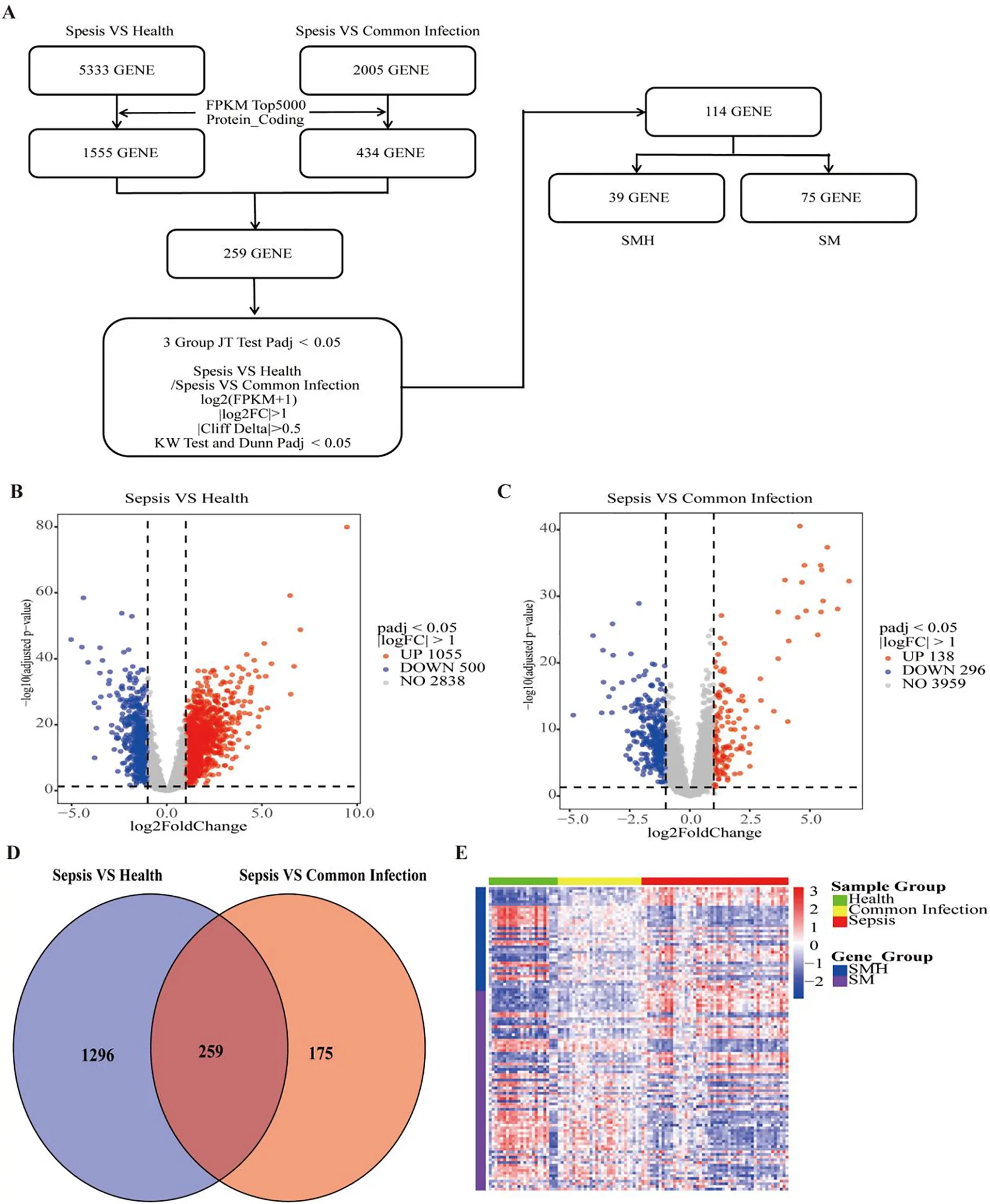
Screening of DEGs in sepsis. **(A)** Schematic diagram showing the workflow for screening the potential diagnostic gene set. **(B, C)** Volcano plots showing the highly expressed DEGs in the sepsis versus healthy control group **(B)** and sepsis versus common infection group **(C)**. **(D)** Venn diagram showing the highly expressed DEGs between the sepsis versus healthy control group and the sepsis versus common infection group. **(E)** Heatmap showing the 114 potential diagnostic genes across the three groups.

Sepsis is triggered by infection, but not all infections inevitably lead to sepsis. Therefore, does comparing the DEGs in peripheral blood immune cells between septic patients and patients with common infections help us understand sepsis pathogenesis and even identify molecular markers with early warning potential for sepsis progression? Based on this rationale, our research group prospectively collected PBMCs from septic patients and performed transcriptome sequencing, including samples from 24 healthy controls, 29 patients with common infections, and 51 septic patients (24, 25). Using this dataset, we attempted to screen for DEGs with diagnostic value for sepsis (**Figure 1A**).

First, we compared the two groups: “sepsis vs. healthy controls” and “sepsis vs. common infections.” A total of 5,333 DEGs were identified in the “sepsis vs. healthy controls” comparison, and 2,005 DEGs were identified in the “sepsis vs. common infections” comparison. Among these, 1,555 and 434 highly expressed DEGs were found in the “sepsis vs. healthy controls” comparison and the “sepsis vs. common infections” comparison, respectively (**Figures 1A–C**). Notably, 259 genes were found to be consistently highly expressed in both comparisons (**Figure 1D**). Further screening using the Jonckheere–Terpstra (JT) test and Kruskal–Wallis (KW) test, combined with |log2FC| and |Cliff’s Delta| for expression abundance, ultimately yielded a set of 114 potential diagnostic genes that exhibited a sustained trend of expression change with disease progression and significant differences between groups (**Figure 1A**). Among these, 39 DEGs were differentially expressed across all three comparisons (“sepsis vs. healthy controls,” “sepsis vs. common infections,” and “common infections vs. healthy controls”), hereafter referred to as SMH genes; the remaining 75 DEGs were differentially expressed in both the “sepsis vs. healthy controls” and “sepsis vs. common infections” comparisons, hereafter referred to as SM genes (**Figure 1E**), which would be applied to further analysis.

### Enrichment analysis of potential diagnostic markers

**Figure 2:**
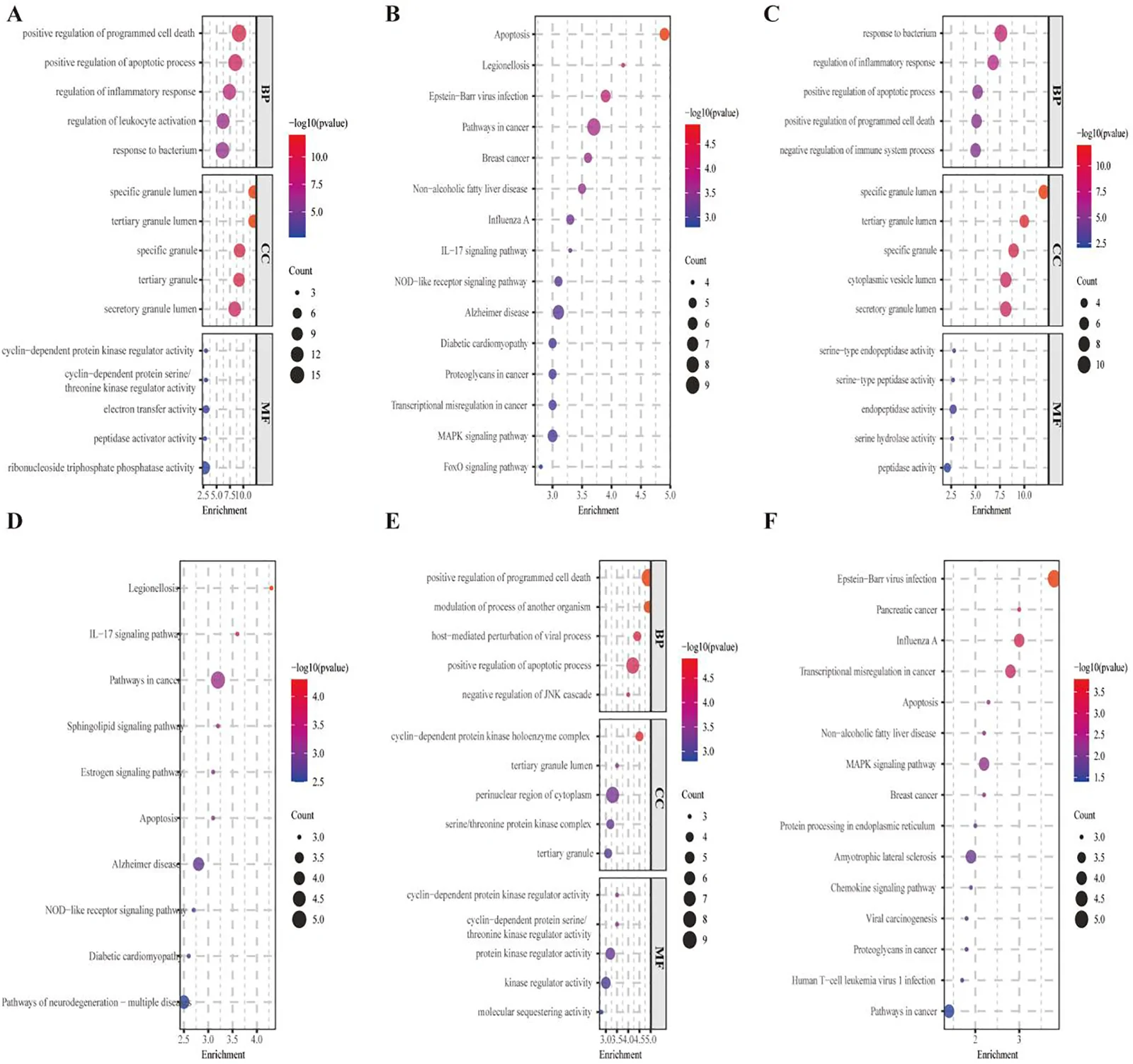
Functional enrichment analysis of DEGs. Bubble plots showing the top 5 BP, CC, MF pathways and significant KEGG pathways for all 114 highly expressed DEGs **(A, B)**, 39 SMH genes **(C, D)**, and 75 SM genes **(E, F)**, respectively.

To investigate the molecular mechanisms underlying sepsis progression and identify potential key nodes that distinguish common infection from sepsis, we performed GO functional enrichment and KEGG pathway analyses on three sets of DEGs: all 114 sepsis-associated genes (**Figures 2A, B**), the 39 SMH genes (**Figures 2C, D**), and the 75 SM genes (**Figures 2E, F**).

Biological processes (BP) enrichment analysis showed that all 114 sepsis-related DEGs and the 39 SMH genes were significantly enriched in pathways related to response to bacteria, regulation of inflammation, positive regulation of apoptosis, and positive regulation of programmed cell death, suggesting their involvement in classical anti-infective immunity and the inflammation-apoptosis cascade (26) (**Figures 2A, C**). In contrast, beyond apoptosis and programmed cell death regulation, the 75 SM genes were specifically enriched in negative regulation of the JNK signaling cascade and perturbation of host-mediated viral processes, indicating that they may play roles in the later stages of sepsis or in more complex host-pathogen interactions (**Figure 2E**).

Cellular components (CC) analysis revealed that the 114 genes and the 39 SMH genes were enriched in specific granules, tertiary granules, and luminal structures of neutrophils, involving degranulation-mediated pathogen clearance and inflammatory amplification (27) (**Figures 2A, C**). In addition to tertiary granules, the SM genes were specifically enriched in cyclin-dependent protein kinase holoenzyme complexes, serine/threonine protein kinase complexes, and perinuclear regions of the cytoplasm, suggesting their involvement in signal transduction, cell cycle regulation, and the spatial assembly of inflammasomes (**Figure 2E**).

Molecular functions (MF) enrichment analysis showed that the 114 genes were mainly enriched in electron transfer activity, peptidase activator activity, and cyclin-dependent protein kinase regulator activity. The 39 SMH genes were significantly enriched in serine-type endopeptidase activity, peptidase activity, and serine hydrolase activity, suggesting that they participate in immune responses and tissue remodeling through peptide bond hydrolysis (**Figures 2A, C**). The 75 SM genes were significantly enriched in protein kinase regulator activity, kinase regulator activity, and molecular chelator activity, indicating that they regulate kinase signaling and the sequestration of active molecules to modulate inflammatory signal amplification and cellular function (**Figure 2E**).

Further study on KEGG pathway analysis demonstrated that all 114 genes and the 39 SMH genes were co-enriched in bacterial infection-related pathways such as NOD-like receptor signaling, IL-17 signaling, and Legionellosis, as well as in the apoptosis pathway, suggesting that these pathways are persistently activated in sepsis, driving cytokine storms and organ damage (**Figure 2B**). In addition, the 39 SMH genes were specifically enriched in pathways related to Alzheimer’s disease, diabetic cardiomyopathy, sphingolipid signaling, and neurodegenerative diseases, implying potential molecular links between sepsis and chronic inflammation or metabolic diseases (**Figure 2D**). In contrast, the 75 SM genes shared with the 114 genes MAPK signaling, pathways in cancer, and virus-related pathways including EBV infection, influenza A, and HTLV-1 infection (**Figure 2F**).

This finding suggests that the SM genes may function as critical molecular switches that delineate common infection from sepsis, driving the onset and progression of multiple organ dysfunction via aberrant inflammatory signaling and immune dysregulation.

### Screening of diagnostic model genes

**Figure 3:**
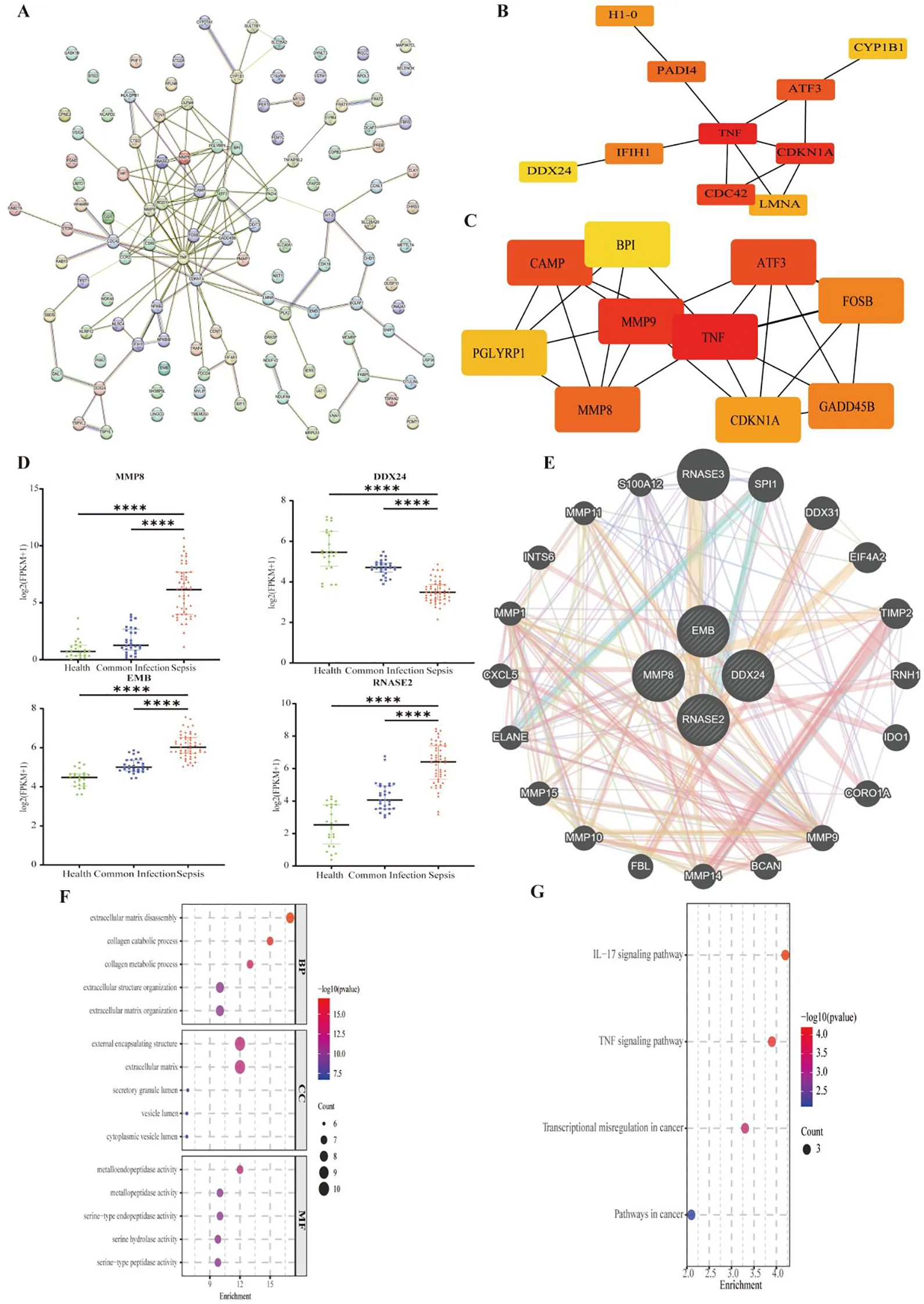
PPI network construction and diagnostic gene screening. **(A)** PPI network of the 114 highly expressed DEGs. **(B, C)** Top 10 hub genes identified by the Betweenness algorithm **(B)** and the MCC algorithm **(C)**. **(D)** Scatter plots showing the expression levels of MMP8, DDX24, EMB, and RNASE2. (Students’ t-test, ****P<0.001) **(E)** PPI network of the four genes constructed using GeneMANIA. **(F, G)** Bubble plots showing the top 5 BP, CC, and MF pathways by GO analysis **(F)** and significant KEGG pathways **(G)** basing on the 24 genes.

To identify genes with robust diagnostic performance and good generalizability for sepsis, we uploaded the 114 DEGs to the STRING database with minimum interaction score threshold of 0.4. This generated a protein–protein interaction (PPI) network comprising 134 edges with an average node degree of 2.35 (**Figure 3A**). The network data were imported into Cytoscape, and the Betweenness and MCC algorithms implemented in the Cytohubba plugin were used to screen for core hub genes. This analysis yielded 16 genes highly relevant to the pathogenesis of sepsis, (**Figures 3B, C**) including TNF, CDKN1A, ATF3, MMP8, DDX24, IFIH1, CDC42, LMNA, CYP1B1, PADI4, H1-0 (H1F0), MMP9, PGLYRP1, CAMP, BPI, FOSB and GADD45B.

We next evaluated the diagnostic performance of these 114 genes. Using R, we calculated AUC values for the comparisons of “sepsis vs. healthy controls” and “sepsis vs. common infections”. The AUC values for the “sepsis vs. healthy controls” comparison were further validated in the public datasets GSE65682 and GSE57065. By comparing the significance of the JT test and the AUC values across different populations, we found that MMP8 and DDX24, the two core hub genes, together with EMB and RNASE2, exhibited higher significance, higher cross-group AUC values, and better generalizability of diagnostic performance compared to the other genes (**Table 1**). Furthermore, the expression abundance of these four genes followed a consistent trend with disease severity (**Figure 3D**).

**Table 1:** The test results and AUC values of individual biomarkers MMP8, DDX24, EMB, and RNASE2.

Dataset	Comparison	MMP8	DDX24	EMB	RNASE2
PBMC RNA-scq	**JT Test Padj**	<2.23E-50	1.57E-15	<2.23E-50	<2.23E-50
	**Sepsis VS Health**	0.989	0.955	0.994	0.987
	**Sepsis VS Common Infection**	0.967	0.952	0.93	0.893
GSE65682	**Sepsis VS Health**	0.956	0.985	0.938	0.968
GSE57065	**Sepsis VS Health**	0.984	0.998	0.957	0.994

*The PBMC RNA-scq dataset is the data for this retrospective study.

JT Test Padj is the corrected P-value for the JT trend test of a single biomarker in the PBMC RNA-scq dataset;Sepsis VS Health: using a single biomarker to distinguish between Sepsis and Health in various datasets; Sepsis VS Common Infection: using a single biomarker to distinguish between Sepsis and Common Infections in the dataset.

To explore the biological functions of these four genes in the onset and progression of sepsis, we submitted them to the GeneMANIA database, which retrieved 20 functionally related genes (**Figure 3E**). GO and KEGG enrichment analyses were performed on these 24 genes. The results revealed significant enrichment in pathways associated with tissue barrier function and inflammation, most notably the TNF and IL-17 signaling pathways (**Figures 3F, G**). These findings indicate that the four candidate genes are closely linked to the early inflammatory response in sepsis.

### Development and performance evaluation of the sepsis diagnostic model

**Figure 4:**
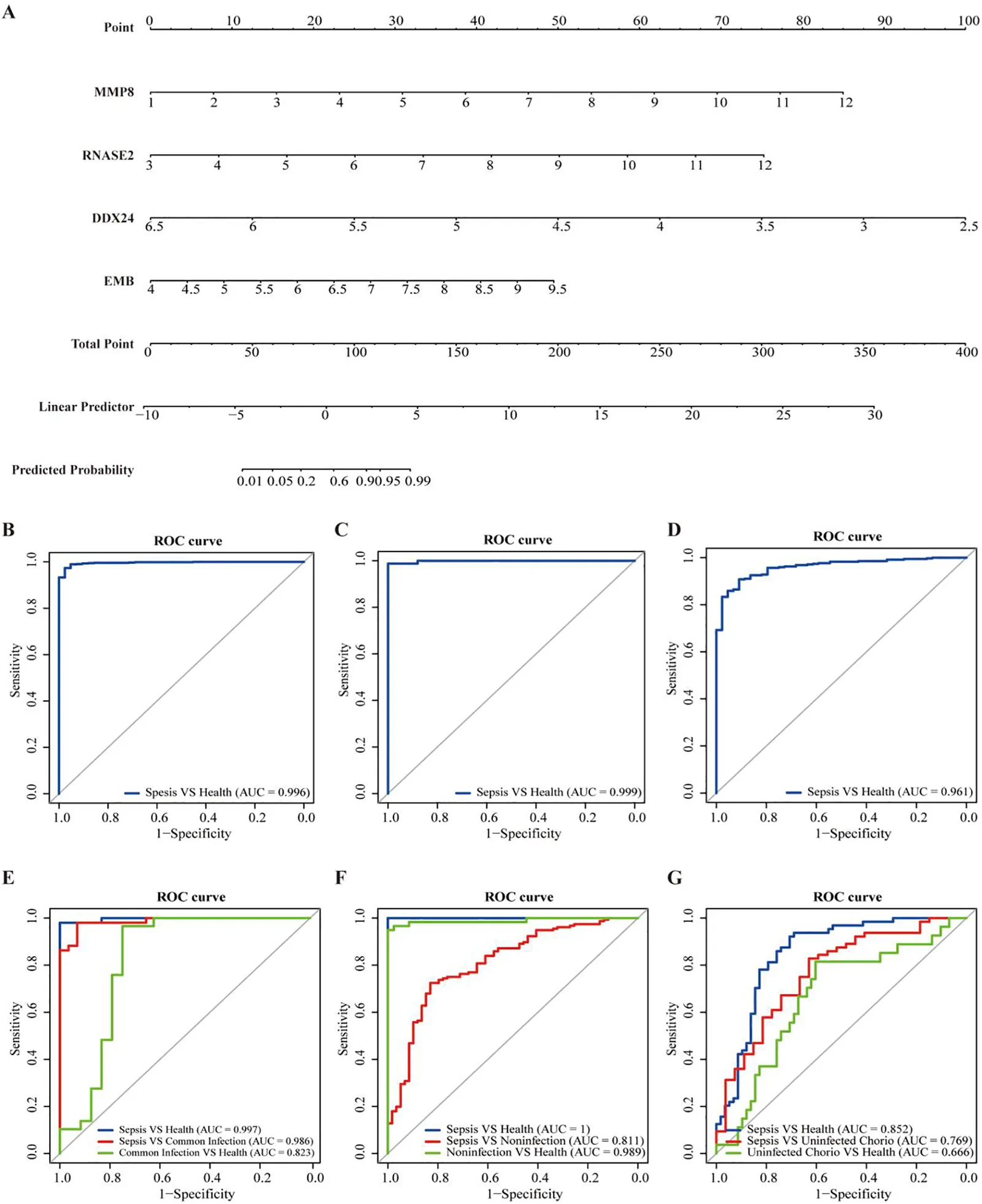
Construction and validation of the model for sepsis diagnosis. **(A)** Construction of a nomogram model for sepsis diagnosis using the selected DEGs. **(B–D)** The ROC curve showing the sensitivity and specificity of the model to distinguish sepsis from healthy control basing on the datasets of GSE65682 **(B)**, GSE57065 **(C)**, and GSE18563 **(D)**, respectively. **(E–G)** The ROC curve showing the sensitivity and specificity of the model to figure out healthy control, common infection and sepsis **(E)**; healthy control, non-infection and sepsis using the public dataset GSE134347 **(F)**; healthy control, uninfected chorio and sepsis using the public dataset GSE69689 **(G)**, respectively.

To assess whether the four screened genes have diagnostic utility for sepsis, we used the public dataset GSE65682 as a training set to construct a LASSO regression model based on these genes. A nomogram was plotted to visualize the model, in which each risk factor is assigned a score; the linear predictor and disease probability are derived from the total score (**Figure 4A**). To evaluate diagnostic performance and generalizability, we plotted ROC curves for three comparisons, which are “sepsis vs. healthy controls”, “sepsis vs. non-sepsis”, and “non-sepsis vs. healthy controls”, in both the training set and multiple validation datasets (**Figures 4B–G**). For each comparison, we calculated the AUC, sensitivity, specificity, accuracy, positive predictive value (PPV) and negative predictive value (NPV), and the optimal cut-off value which is corresponding to the linear predictor in the nomogram (**Table 2**, **Supplementary Table 1**).

**Table 2:** Validation of diagnostic efficacy and accuracy for the diagnostic model.

Dataset	Comparison	AUC	Sensitivity	Specificity	Cut-off	BSS	Calibration Intercept	Calibration Slope
GSE65682	**Sepsis VS Health**	0.996	0.974	0.976	2.167	0.803	-0.13	1.198
PBMC RNA-scq	**Sepsis VS Health**	0.997	0.98	1	0.529	0.897	-0.427	1.743
	**Sepsis VS Common Infection**	0.986	0.98	0.931	0.774	0.754	-1.394	1.018
	**Common Infection VS Health**	0.823	0.966	0.75	-4.293	-0.464	1.992	0.471
GSE57065	**Sepsis VS Health**	0.999	0.988	1	1.087	0.918	-1.265	1.721
GSE185263	**Sepsis VS Health**	0.961	0.908	0.909	0.837	0.48	0.786	0.879
GSE134347	**Sepsis VS Health**	1	1	1	0.852	0.964	-22.1	25.675
	**Sepsis VS Noninfection**	0.811	0.724	0.831	7.607	-0.209	-1.678	0.373
	**Noninfection VS Health**	0.989	0.949	1	0.139	0.877	0.016	1.829
GSE69686	**Sepsis VS Health**	0.852	0.922	0.707	4.002	-0.393	-2.509	0.461
	**Sepsis VS Uninfected Chorio**	0.769	0.828	0.63	5.328	-0.239	-1.461	0.355
	**Uninfected Chorio VS Health**	0.666	0.815	0.603	2.856	-1.34	-1.416	0.16

*Noninfection: Patients who were not infected and were admitted to the ICU 24 hours later.

*Uninfected Chorio: Newborns who were not infected but had been exposed to chorioamnionitis. In this study, they were classified into the patient group whose condition was between sepsis and healthy controls.

Sepsis VS Health: Using the model to distinguish between Sepsis and Health in various datasets; Sepsis VS Common infection: using the model to distinguish between Sepsis and Common Infection in the dataset; Common infection VS Health: using the model to distinguish between Common infection and Health in the dataset; Sepsis VS Noninfection: using the model to distinguish between Sepsis and Noninfection in the dataset; Noninfection VS Health: using the model to distinguish between Noninfection and Health in the dataset; Sepsis VS Uninfected Chorio: using the model to distinguish between Sepsis and Uninfected Chorio in the dataset; Uninfected Chorio VS Health: using the model to distinguish between Uninfected Chorio and Health in the dataset.

In the training set, the model achieved an AUC of 0.996, a sensitivity of 0.974, a specificity of 0.976, an accuracy of 0.974, a PPV of 0.999, and a NPV of 0.672, indicating good diagnostic performance (**Table 2**, **Supplementary Table 1**). We then validated the model using the public datasets GSE57065, GSE185263, GSE134347 and our own retrospective cohort. The results showed an AUC range of 0.811-1, sensitivity 0.724-1, specificity 0.75-1, accuracy 0.753-1, PPV 0.824-1, and NPV 0.533-1. In the “sepsis vs. healthy controls” comparison specifically, the AUC ranged from 0.961 to 1, sensitivity from 0.908 to 1, specificity from 0.909 to 1, accuracy from 0.908 to 1, PPV from 0.988 to 1, and NPV from 0.556 to 1(**Table 2**, **Supplementary Table 1**). These results demonstrate strong generalizability and effective differentiation among sepsis patients, healthy controls, and patients with intermediate disease severity.

We further evaluated the model’s performance in neonatal sepsis using the GSE69686 dataset. The model yielded an AUC range of 0.666-0.852, sensitivity 0.815-0.922, specificity 0.603-0.707, accuracy 0.671-0.82, PPV 0.489-0.776, and NPV 0.607-0.891(**Table 2**, **Supplementary Table 1**). These findings suggest that the model also has some diagnostic value for neonatal sepsis.

### Validation of diagnostic model accuracy

Following the initial evaluation of diagnostic performance, we further assessed the model’s predictive accuracy using the Brier Skill Score (BSS) and calibration curves.

**Figure 5:**
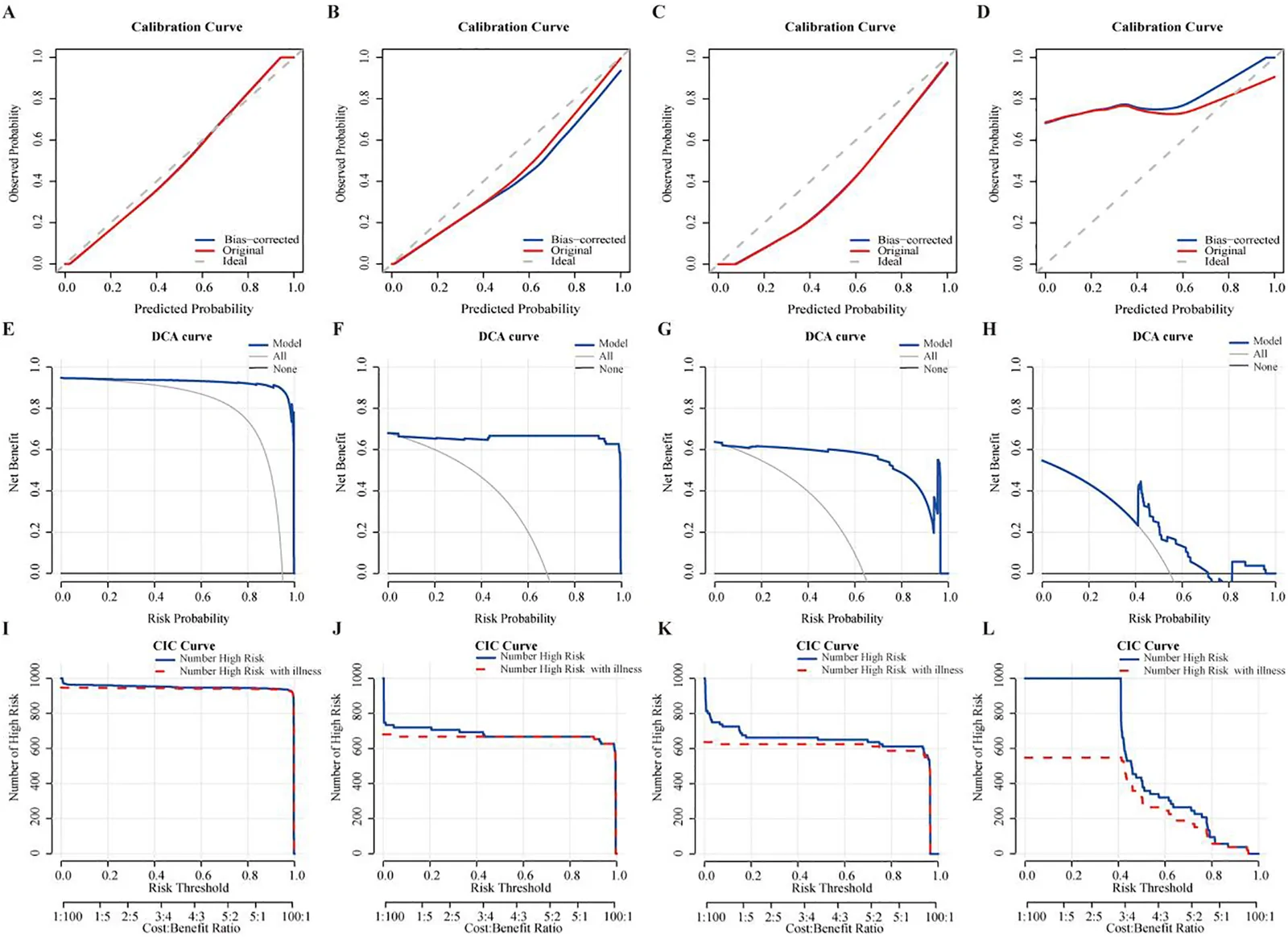
Accuracy validation and clinic utility assessment of the model. **(A)** The calibration curve showing the predictive accuracy of the model to distinguish sepsis from healthy control basing on the datasets of GSE65682. **(B–D)** The calibration curve showing the predictive accuracy of the model to figure out “healthy control and sepsis” **(B)**, “common infection and sepsis” **(C)**, and “healthy control and common infection” **(D)**, respectively. **(E)** The DCA curve showing the clinical utility of the model to distinguish sepsis from healthy control basing on the datasets of GSE65682. **(F–H)** The DCA curve showing the clinical utility of the model to figure out “healthy control and sepsis” **(F)**, “common infection and sepsis” **(G)**, and “healthy control and common infection” **(H)**, respectively. **(I)** The CIC curve showing the clinical utility of the model to distinguish sepsis from healthy control basing on the datasets of GSE65682 in a reference population. **(J–L)** The CIC curve showing the clinical utility of the model to figure out “healthy control and sepsis” **(J)**, “common infection and sepsis” **(K)**, and “healthy control and common infection” **(L)** in a reference population, respectively.

In the training set, the BSS was 0.803 (**Table 2**), indicating that the model’s probabilistic prediction performance was substantially improved over the reference model, reflecting good discriminative ability and calibration potential, and supporting its high diagnostic value. In the non-neonatal validation sets, BSS values were greater than 0 across all comparison groups (range 0.48–0.964), with only two exceptions: the “common infection vs. healthy controls” group in the retrospective data (-0.464) and the “sepsis vs. non-infected patients” group in GSE134347 (-0.209). This suggests that in these specific retrospective subgroups, the model did not provide predictive information superior to the reference probability, which may be related to sample characteristics or data bias; nevertheless, the majority of results indicate good overall predictive accuracy. Concurrently, the corresponding calibration curves showed that most of the original and bias-corrected curves closely approximated the 45° ideal line (**Figures 5A–D**, **Supplementary Figures 1–3**), further confirming that the model’s predicted probabilities were well calibrated with the observed frequencies, supporting its high diagnostic value.

We also constructed a comparison model using four hub genes (PGLYRP1, MMP9, CYP1B1, BPI) identified from the individual marker diagnostic efficacy screening (**Supplementary Table 2**). This hub-gene model achieved a training-set AUC of 0.988 and a BSS of 0.625; in validation sets, the AUC ranged from 0.641 to 0.999 and the BSS from -3.417 to 0.413 (**Supplementary Table 3**). All metrics were inferior to those of our model, indicating that the diagnostic model based on our four candidate genes has superior predictive accuracy and diagnostic performance.

In the neonatal population, the model’s BSS below 0, and the calibration curve deviated substantially from the ideal line, indicating poor predictive accuracy. Together with the AUC results in this population, these findings suggest that the model is not suitable for diagnosing neonatal sepsis and offers only limited suggestive value (**Supplementary Figure 4**).

### Assessment of model clinical utility

To evaluate the clinical utility of our model, we performed decision curve analysis (DCA) and clinical impact curve (CIC) analysis. DCA compares the net benefit of a model across different threshold probabilities against two extreme strategies: “intervene for all” and “intervene for none” (28). CIC further illustrates, within a reference population, the number of individuals classified as high-risk by the model and the number of true positives among them at each threshold (29). The threshold reflects the cost-benefit trade-off; for example, a ratio of 1:100 means that treating one patient comes at the expense of 100 individuals with mild illness or healthy status.

In the training set (**Figures 5E, I**), the model provided clinical utility when the threshold exceeded 0.3, corresponding to a cost-benefit ratio of 2:5. In the retrospective validation cohort (**Figures 5F, J**), for the comparison of “sepsis vs. healthy controls”, clinical utility was observed at thresholds above 0.4, with a cost-benefit ratio of 3:4. For “sepsis vs. common infection” (**Figures 5G, K**), the model demonstrated clinical utility across a threshold range of approximately 0.2-0.9, with cost-benefit ratios ranging from 1:5 to 5:1. For “common infection vs. healthy controls” (**Figures 5H, L**), clinical utility was observed across a threshold range of approximately 0.4-0.7, with cost-benefit ratios ranging from 3:4 to 5:2.

### CIBERSORT immune infiltration analysis and correlation with diagnostic genes

Previous enrichment analyses suggested that the four diagnostic genes are associated with inflammatory and immune processes. To explore this further, we used CIBERSORT to analyze immune infiltration across samples.

**Figure 6:**
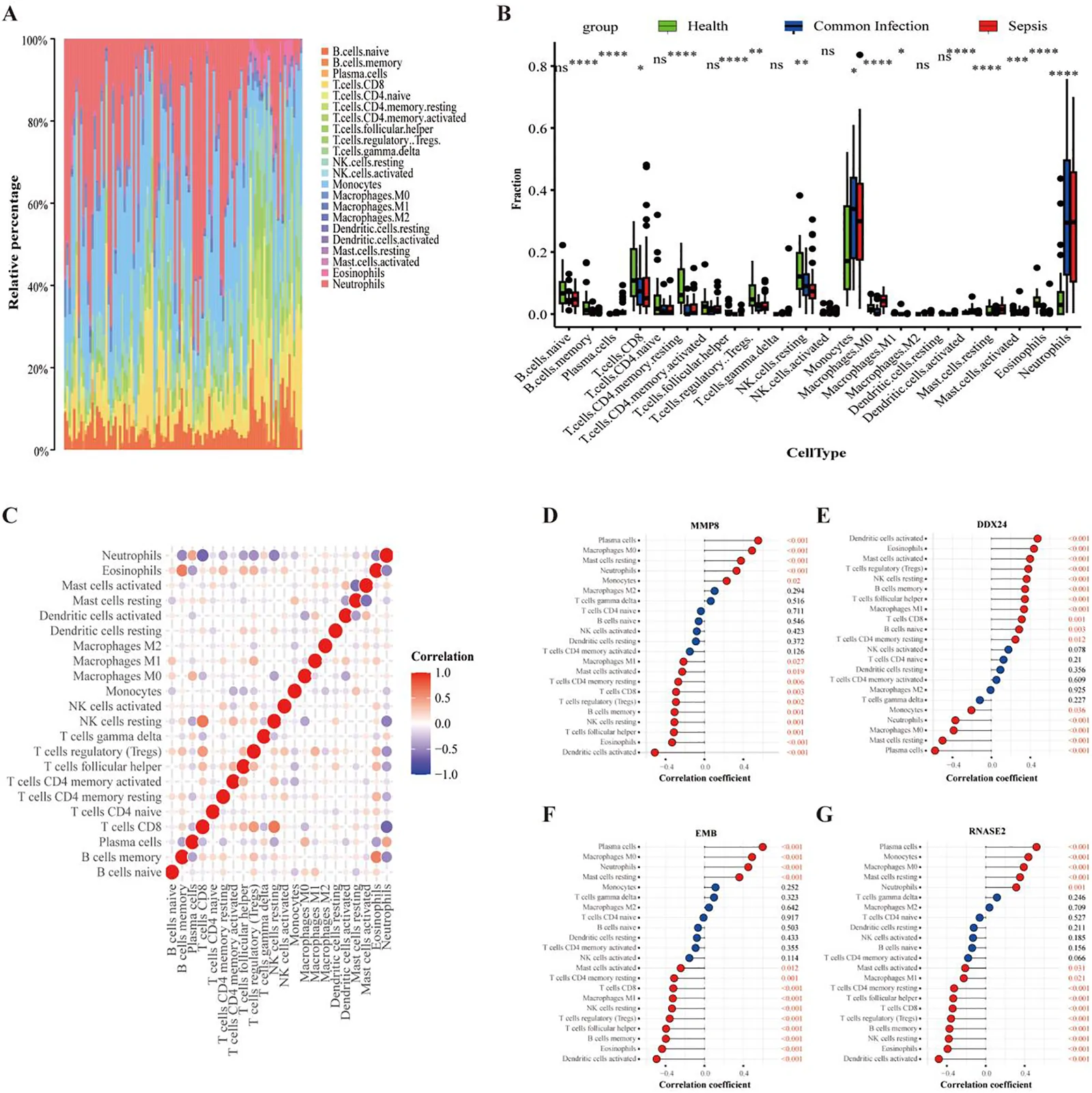
Immune infiltration landscape of sepsis. **(A)** Diagram showing the proportions of 22 immune cell types using the transcriptomic dataset. **(B)** Bar graph showing the proportions of 22 immune cells in indicated group. **(C)** Bubble plot showing the correlation relationship of the 22 immune cell types. **(D–G)** Bubble plots showing the correlation relationship of MMP8, DDX24, EMB, and RNASE2 with immune cell infiltration. *p < 0.05, **p < 0.01, ***p < 0.001, ****p < 0.0001.

Several patterns emerged (**Figures 6A, B**). The abundance of CD8^+^T cells and resting NK cells negatively correlated with infection severity. Healthy controls had higher abundances of resting memory CD4^+^T cells and regulatory T cells (Tregs) than patients with common infections or sepsis. Monocyte abundance first increased and then decreased with infection severity. In addition, neutrophil abundance was obviously elevated in patients with common infections and sepsis. Similar observation was obtained from quanTIseq analysis (**Supplementary Figure 5**), indicating a substantial release of low-density neutrophils, which is consistent with the previous studies (30–32). Furthermore, the increase of neutrophil-associated genes, such as MMP8 and RNASE2, indicated the regulation of neutrophils on septic immune response (33).

Correlation analysis (**Figure 6C**) showed positive associations among CD8^+^T cells, resting memory CD4^+^T cells and Tregs, whereas these three cell types were negatively correlated with monocytes. Furthermore, CD8^+^T cells, resting memory CD4^+^T cells, Tregs and monocytes were all negatively correlated with neutrophils.

We next examined correlations between the diagnostic genes and specific immune cell subsets (**Figures 6D–G**). MMP8, EMB and RNASE2 were negatively correlated with CD8^+^T cells, resting NK cells, resting memory CD4^+^T cells and Tregs, but positively correlated with neutrophils. In addition, both MMP8 and RNASE2 showed positive correlations with monocytes. By contrast, DDX24 was positively correlated with CD8^+^T cells, resting NK cells, resting memory CD4^+^T cells and Tregs, but negatively correlated with monocytes and neutrophils.

### GSEA analysis of diagnostic markers

To explore the potential functional mechanisms of the four diagnostic genes in infection and sepsis, we performed GSEA-KEGG enrichment and immune signature analyses based on high- and low-expression groups.

**Figure 7:**
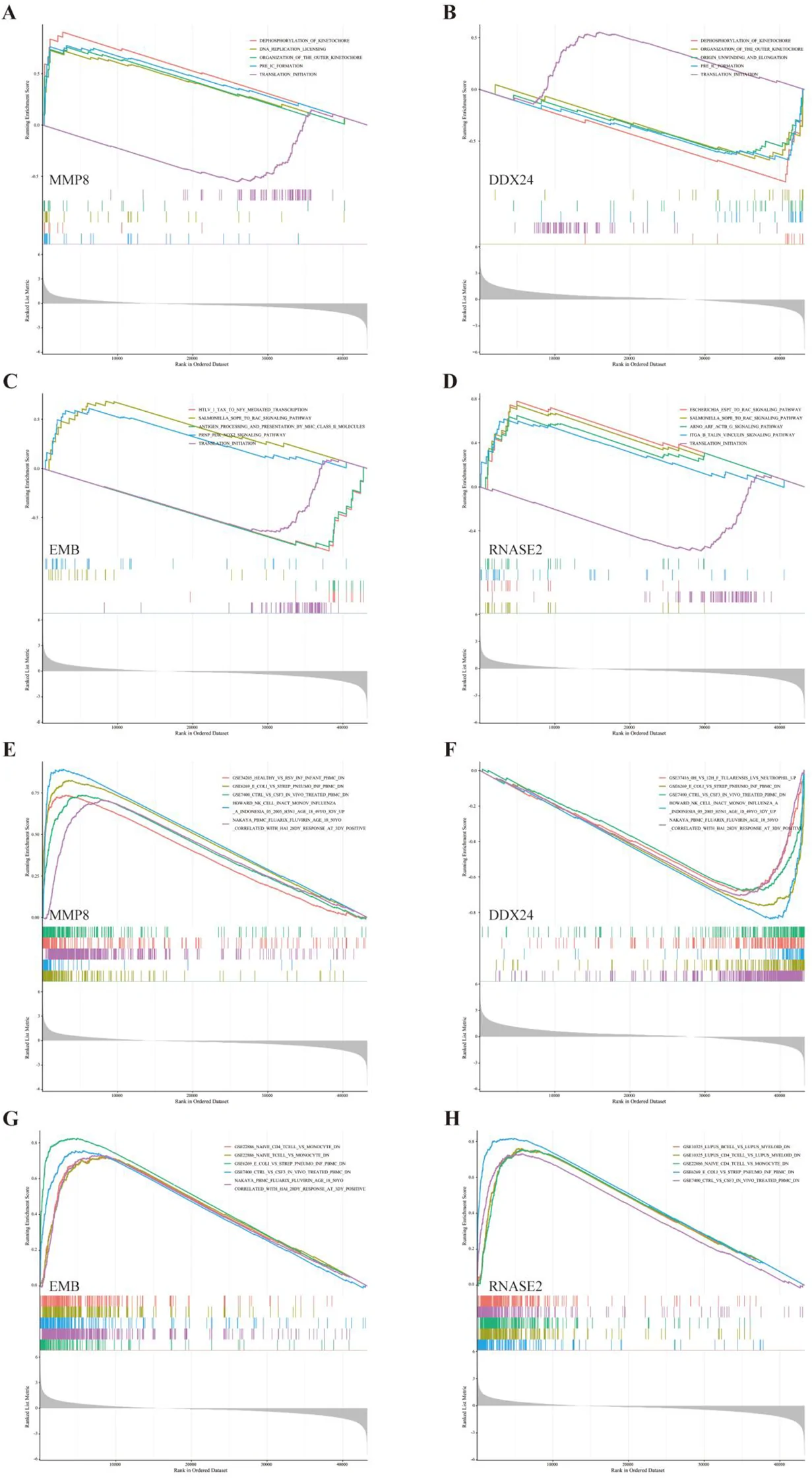
GSEA analysis on the diagnostic genes. **(A–D)** GSEA-KEGG pathway analyses for MMP8, DDX24, EMB, and RNASE2. **(E–H)** GSEA of immune signatures for MMP8, DDX24, EMB, and RNASE2.

MMP8 and DDX24 were jointly involved in regulating immune complex formation, cell cycle progression, DNA replication and mitotic processes, displaying an antagonistic pattern (**Figures 7A, B**). EMB and RNASE2 were primarily enriched in pathways related to pathogenic infection, oxidative stress, cytoskeletal adhesion and immune cell adhesion (**Figures 7C, D**). In addition, all four genes participated in translation initiation.

Immune signature analysis revealed the following patterns. MMP8, EMB and RNASE2 showed high-expression enrichment during bacterial (e.g., E. coli, S. pneumoniae, F. tularensis) and viral (e.g., H5N1, RSV) infections, participating in acute infection and myeloid cell activation. By contrast, DDX24 consistently showed negative enrichment, with low expression during pathogen infection, neutrophil/monocyte inflammatory activation and NK cell suppression, forming an antagonistic regulatory pattern with MMP8 in infection immunity. High expression of MMP8 and EMB, together with low expression of DDX24, co-enriched in pathways related to early immune responses to influenza vaccine and late antibody responses, suggesting that these three genes collectively contribute to vaccine-induced adaptive immune activation. High expression of EMB and RNASE2 was significantly enriched in monocyte/myeloid cell signature pathways, deviating from naive T cell and B cell lineages and exhibiting a myeloid bias. (**Figures 7E–H**) Furthermore, all four diagnostic genes co-enriched in immune pathways associated with CSF3 colony-stimulating factor intervention.

## Discussion

With its substantial mortality burden, sepsis is a critical threat to global health security (34, 35). The core pathogenesis of sepsis lies in a severe dysregulation of the host immune response to infection, characterized by the coexistence and dynamic evolution of hyperinflammation and immunosuppression during the disease course (36, 37). Infection leads to strong activation of monocytes/macrophages and neutrophils by pathogen-associated molecular patterns (PAMPs) and damage-associated molecular patterns (DAMPs), promoting the release of large amounts of pro-inflammatory cytokines and abnormal activation of the complement and coagulation systems (38). While these responses aim to clear pathogens, they also exacerbate tissue damage and multi-organ dysfunction caused by the infection. The emergence of an immunosuppressive state, characterized by extensive apoptosis and functional exhaustion of lymphocytes (especially T cells and B cells) (39, 40), abnormal expansion of myeloid-derived suppressor cells (MDSCs) (41, 42), sustained downregulation of HLA-DR expression on monocyte surfaces (43), and upregulation of negative co-stimulatory molecules such as PD-1 and TIM-3 (44–47), severely impairs the host’s ability to clear pathogens, predisposing patients to secondary infections and leading to disease deterioration. The aforementioned immune status characteristics were partially corroborated in the enrichment and immune infiltration analyses of this study. Moreover, the virus-related pathways identified in the GO and KEGG analyses of SM genes suggest an increased risk of disease progression from common infection to sepsis or exacerbation of sepsis, providing a rationale for screening early diagnostic molecular markers for sepsis from genes with altered expression in peripheral blood immune cells of septic patients.

Sepsis is highly heterogeneous. Comparing DEGs in peripheral blood immune cells between septic patients and healthy controls yields thousands of candidate genes (**Figure 1A**). In this study, we additionally included a group of patients with common infections who did not progress to sepsis. This approach not only facilitates a deeper understanding of the immune response in sepsis but also helps exclude the influence of normal gene expression changes during the immune response to common infections, thereby aiding the screening of high-risk genes involved in sepsis progression. Through differential expression analysis and screening based on expression abundance changes, we preliminarily identified 114 sepsis-infection signature genes that exhibited persistent changes with disease progression and possessed certain diagnostic efficacy. After cross-validation across multiple datasets and populations, four genes highly relevant to sepsis diagnosis were ultimately identified: MMP8, DDX24, EMB, and RNASE2.

MMP8, also known as neutrophil collagenase or collagenase-2, is expressed in various immune cells and participates in sepsis-related inflammatory responses (48–50). Our study found that MMP8 expression levels increased with infection severity. CIBERSORT and GSEA analyses showed that it is highly expressed in monocytes/neutrophils and enriched in pathogen infection pathways, consistent with a report by Kong et al. that the AUC of MMP8 for diagnosing septic shock was 0.932 (51). Functionally, Lu et al. found that knockdown of MMP8 reduced mortality in septic mice (50), suggesting that it is not only a diagnostic marker but also a potential therapeutic target. Notably, MMP8 and DDX24 exhibited an antagonistic expression pattern, suggesting they may play opposing roles in the immune regulation of sepsis.

DDX24 is an RNA helicase involved in innate immunity (52, 53), vascular malformations (54), and anti-oxidative apoptosis (55). Our study found that DDX24 expression decreased with infection severity, supporting previous research findings (56). Furthermore, DDX24 was found to be highly correlated with T cells and NK cells, consistent with a recent report by Zhang et al. (57). Its antagonistic relationship with MMP8 further suggests that DDX24 may serve as a potential therapeutic target for sepsis, although the exact mechanism requires further investigation.

EMB encodes an immunoglobulin superfamily transmembrane glycoprotein that is significantly dysregulated in sepsis (58). GSEA analysis showed that high EMB expression is mainly enriched in pathways related to pathogenic infection, oxidative stress, cytoskeletal adhesion, and immune cell adhesion, all of which are important components of the immunopathology of sepsis. Notably, our study found that high EMB expression was negatively correlated with CD8^+^T cells, resting memory T cells, and Treg cells, exhibiting a myeloid cell bias. This finding is inconsistent with a report by Yang et al. showing high EMB expression in CD4^+^/CD8^+^T cells (59). However, that study also indicated that EMB is not essential for T cell development, effector function, or memory formation, which aligns with our finding that EMB was not identified as a sepsis hub gene.

RNASE2 is a secretory protein expressed in leukocytes with antiviral activity against single-stranded RNA viruses (60–62). Our GSEA immune signature analysis also showed that high RNASE2 expression was enriched in pathways related to pathogenic infection, early immune response to influenza vaccine, and late antibody responses to influenza. Multiple studies support RNASE2 as a diagnostic marker for sepsis. For example, Chen et al. combined microarray data with single-cell RNA sequencing and found that its AUC for diagnosing sepsis and infective endocarditis reached 0.91-1 (63). Recently, Meng et al. used cross-validation with LASSO regression and random forest to confirm RNASE2 as a core diagnostic gene, with their model achieving an AUC as high as 0.903 in validation sets (64).

Based on these four genes and using the GSE65682 dataset as the training set, we constructed a LASSO regression model. The model achieved an AUC of 0.996 and a BSS of 0.803 for distinguishing sepsis from healthy controls. Across multiple validation set populations, the AUC ranged from 0.811 to 1, and BSS were mostly above 0, indicating good discrimination and calibration (**Figure 4**).

It is worth noting that most studies constructing sepsis diagnostic models tend to use sepsis hub genes, believing that hub genes have better diagnostic capability for indicating sepsis status. However, EMB and RNASE2 used in our model are not traditionally considered sepsis hub genes (65–67). To validate the rationale for our model construction strategy, we selected four hub genes (PGLYRP1, MMP9, CYP1B1, BPI) whose individual diagnostic efficacy was second only to MMP8 and DDX24 in our gene screening to construct a comparison model (**Supplementary Table 2**). The results (**Supplementary Table 3**) showed that this hub-gene model achieved an AUC of 0.988 and a BSS of 0.625 in the training set, and an AUC range of 0.641-0.999 and BSS range of -3.417 – 0.413 in validation sets, with all metrics inferior to those of our model. This finding suggests that the diagnostic efficacy of an individual gene is not equivalent to its predictive contribution as part of a multivariate model; high differential expression or strong individual correlation does not necessarily translate into superior combined diagnostic performance. Therefore, screening for diagnostic markers should consider both the biological relevance of genes and their synergistic contributions within a multivariate model.

On this basis, we used DCA and CIC curve analyses to further confirm that our model provides clinical net benefit in different comparison scenarios (**Figure 5**). However, in the validation for neonatal sepsis, the model’s AUC (0.666-0.852) and calibration (BSS < 0) were suboptimal, suggesting that the model is not suitable for the neonatal population and its application should be strictly limited to adults or specific age groups. We speculate that this limitation may arise from the immaturity of the neonatal immune system, rendering immune response-based molecular indicators insufficiently effective for diagnosing neonatal sepsis.

To further improve the sepsis diagnostic model and enhance its diagnostic and clinical utility, we propose the following directions for improvement: (1) The retrospective nature and limited sample sizes of the validation datasets, along with the use of early versions of sepsis diagnostic criteria in some datasets, have led to substantial heterogeneity between datasets. Meanwhile, the current external validation datasets lack sufficient samples from general infection or non-infectious critical illness. Therefore, the model’s ability to discriminate between sepsis and common infection has only been preliminarily evaluated in our in-house discovery cohort and has not yet received independent external validation. Well-designed prospective trials with larger sample sizes incorporating multi-omics data analysis could help address this issue. (2) Only a single method (LASSO regression) was used to construct the diagnostic model. Incorporating multiple machine learning algorithms such as random forest and support vector machines could further optimize the model to explore more advantageous sepsis diagnostic models. (3) Further laboratory research, animal experiments, and clinical trials are needed to elucidate the regulatory roles and specific mechanisms of the aforementioned molecular markers in the immune response during sepsis, thereby exploring their potential for the early diagnosis and treatment of sepsis. A prospective cohort study specifically collecting blood samples from patients with sepsis and common infections are currently conducted by our group, which may help to provide robust data for further clinical validation and translation.

## Materials and methods

### Data collection and processing

The bulk-RNA sequencing data on peripheral blood mononuclear cells from patients collected by our research group was applied for retrospective analysis, which include 24 healthy controls, 29 common infectious patients and 51 sepsis patients (24, 25). The study was approved by the Biomedical Ethics Committee of West China Hospital, Sichuan University (Approval No. 2020641). And the dataset was available at https://ngdc.cncb.ac.cn/gsa-human/browse/HRA002988.

To assess the diagnostic model and validate the findings, multiple public datasets were used in this study. Specifically, the datasets including sepsis patients and healthy controls or additionally containing the patient groups with conditions intermediate between sepsis and health were searched from the NIH GEO database and ultimately the datasets numbered GSE69682, GSE57065, GSE185263, GSE134347, and GSE69686 were selected for further analysis. The matrix data (.txt format file) of microarray datasets (GSE69682, GSE57065, GSE134347, and GSE69686) were downloaded from the GEO database through standard pipelines and applied to probe annotation and deduplication before analysis. What’s more, the FPKM value of the bulk-RNA sequencing dataset (GSE185263) was also downloaded from GEO database and followed by log2 transformation, annotation, and deduplication. All operations were performed on R platform (**Table 3**).

**Table 3:** The information of the relevant data set for this study.

Dataset	People	Samples size	Samples size	Samples size	Data type	Source type	Function
PBMC RNA-scq	Adult	Sepsis(n=51)	Health(n=24)	Common Infection(n=29)	RNA-scq	PBMC	Validation set, screening dataset
GSE65682	Adult	Sepsis(n=760)	Health(n=42)		Microarray	Whole blood	Training set, filtered dataset
GSE57065	Adult	Sepsis(n=82)	Health(n=25)		Microarray	Whole blood	Validation set, screening dataset
GSE185263	Adult	Sepsis(n=348)	Health(n=44)		RNA-scq	Whole blood	Independent validation set
GSE134347	Adult	Sepsis(n=156)	Health(n=83)	Noninfection(n=59)	Microarray	Whole blood	Independent validation set
GSE69686	Neonate	Sepsis(n=64)	Health(n=58)	Uninfected Chorio(n=27)	Microarray	Whole blood	Independent validation set

### Screening of target genes

In this study, the DESeq2 method was used to identify differentially expressed genes (DEGs) through pairwise comparisons among three groups: sepsis, common infection, and healthy control. DEGs were defined using an adjusted P-value < 0.05 and |log2FC| > 1. The RNA-seq data used for this analysis were generated by Novogene through its RNA-seq sequencing service.

To screen for potential diagnostic markers showing monotonic trends with increasing infection severity, protein-coding genes were first extracted from the top 5,000 genes ranked by descending sum of FPKM values. The intersection of genes that were significantly differentially expressed in both the “sepsis vs. healthy control” and “sepsis vs. common infection” comparisons was retained. For this gene set, expression abundances after log2 (FPKM + 1) transformation were calculated for each sample. Based on the increasing infection severity (healthy control, common infection, sepsis), the JT trend test was performed using the R package “clinfun” (68) to identify genes with monotonically increasing or decreasing expression trends. Meanwhile, the KW test from the “rstatix” package, followed by Dunn’s post-hoc test, was used to assess overall differences among groups and pairwise comparisons. To quantify the biological and clinical significance of differences, |log2FC| and |Cliff’s Delta| between groups were calculated using the “effsize” package. Given the deterministic relationship between |Cliff’s Delta| and the area under the ROC curve (AUC), where AUC = (|Cliff’s Delta| + 1)/2, a |Cliff’s Delta| > 0.5 is equivalent to AUC > 0.75, indicating that the gene possesses at least moderate diagnostic discrimination ability.

The criteria for selecting the target gene set were as follows: (1) In the DESeq2 differential analyses of both “sepsis vs. healthy control” and “sepsis vs. common infection”, adjusted P-value < 0.05 and |log2FC| > 1; (2) JT test adjusted P-value < 0.05; (3) KW test and Dunn’s post-hoc test adjusted P-values < 0.05 for both the “sepsis vs. healthy control” and “sepsis vs. common infection” comparisons; (4) Based on log2(FPKM + 1) expression abundance, |log2FC| > 1 for both the “sepsis vs. healthy control” and “sepsis vs. common infection” comparisons, and |Cliff’s Delta| > 0.5 (AUC > 0.75).

Additionally, the R package “venn” was used to draw a Venn diagram showing the intersection of genes that were significantly differentially expressed in both DESeq2 comparisons among the top 5,000 FPKM protein-coding genes. The “ggplot2” package was used to draw volcano plots of DEGs from the top 5,000 FPKM genes for the “sepsis vs. healthy control” and “sepsis vs. common infection” comparisons, respectively. The “pheatmap” package was used to plot a heatmap of the screened target gene set.

### Functional enrichment analysis of DEGs

To explore the potential biological significance of the target genes, we performed Gene Ontology (GO) and Kyoto Encyclopedia of Genes and Genomes (KEGG) pathway analyses on the screened sepsis-related genes using Metascape(https://metascape.org/gp/index.html#/main/step1) (69). Metascape is a comprehensive bioinformatics tool that integrates over 40 biological databases and provides robust pathway enrichment analysis. Through this analysis, we annotated the BP, CC, and MF of the target genes, and identified biological pathways potentially implicated in disease processes. The analysis parameters were set as follows: minimum overlap = 3, P-value cutoff = 0.05, and minimum enrichment = 1.5. The enrichment results were visualized using bubble plots generated with the “ggplot2” package.

### Protein-protein interaction network analysis and hub gene identification

We constructed a PPI network for the sepsis-related gene set using the STRING database (https://string-db.org/) (70), with the minimum required interaction score threshold set to 0.4. After constructing the network, the corresponding.csv file was downloaded and imported into Cytoscape software (71). The top 10 hub genes were identified using two algorithms, Betweenness and MCC, from the Cytohubba plugin. After determining the genes to be used for constructing the diagnostic model, the GeneMANIA database (https://genemania.org/) (72) was further employed to predict genes interacting with these diagnostic genes, and a PPI network diagram was plotted.

### Screening of diagnostic genes across multiple datasets

To more accurately identify genes for sepsis diagnosis, we calculated the AUC values of the previously screened sepsis-related genes in the “sepsis vs. healthy control” and “sepsis vs. common infection” comparisons, as well as in the public datasets GSE65682 and GSE57065, using the “pROC” package. This allowed us to evaluate the diagnostic performance of individual genes across the two group comparisons. By comparing the AUC values of each gene across different populations and datasets, and integrating the adjusted P-values from the JT test, we ultimately selected the genes with the most significant monotonic changes with increasing infection severity and better diagnostic performance for constructing a sepsis diagnostic model.

### Diagnostic model construction

LASSO regression, an extension of linear regression that incorporates an L1 regularization term into the loss function to perform variable selection and coefficient shrinkage, is well suited for addressing multicollinearity, high-dimensional data, and overfitting (73). Therefore, after identifying the candidate genes, we applied LASSO regression to construct a diagnostic model for sepsis. In this analysis, we used the “glmnet” package in R and performed five-fold cross-validation via the cv.glmnet function to select the regularization parameter λ that minimized the cross-validation error (lambda.min). The relevant parameter settings, including alpha = 1 (LASSO penalty) and family = “binomial” (binary logistic regression), were specified in the code, which has been uploaded along with the manuscript. Meanwhile, given the relatively small sample sizes in each group of the retrospective analysis dataset, and the fact that patients with common infections present with an intermediate severity between sepsis patients and healthy controls, we selected the public dataset GSE65682 as the training set. The retrospective dataset and the other public datasets (GSE57065, GSE185263, GSE134347, and GSE69686) were used as validation sets. A nomogram was plotted using the “rms” package to visualize the diagnostic model based on the training set. ROC curves were generated to calculate the AUC, specificity, sensitivity, and other metrics to evaluate the model’s diagnostic performance. Calibration curves were plotted, and the Brier score was calculated to quantify the model’s diagnostic accuracy (74). Finally, DCA and CIC were plotted using the “rmda” package to assess the clinical net benefit of the diagnostic model across different populations.

### Immune infiltration analysis

CIBERSORT analysis was performed on each sample to estimate the proportions of various immune cell types. CIBERSORT is a bioinformatics tool that infers the abundances of different cell types in complex tissues from transcriptome data using a support vector regression (SVR) algorithm through a deconvolution approach (75). According to the information provided on the CIBERSORT website (https://cibersortx.stanford.edu/), LM22 is a gene expression signature matrix containing 22 distinct immune cell types. The relevant data and analysis code were downloaded from the GitHub platform (https://github.com/Moonerss/CIBERSORT/blob/main/R/CIBERSORT.R), and the proportions of the 22 immune cell types in each sample were estimated using R language. The results were visualized using the R packages “ggpubr” and “ggplot2”. Additionally, Spearman’s non-parametric test was used to evaluate the correlations between the diagnostic model genes and immune cell infiltration. To support the CIBERSORT analysis results, we also performed quanTIseq immune infiltration analysis (76, 77) to further characterize the immune landscape of the samples.

### Gene set enrichment analysis

To explore the potential biological functions of the diagnostic genes, samples in the retrospective analysis dataset were reclassified into high-expression and low-expression groups based on the median expression abundance of the diagnostic genes. Pathway-related gene sets were downloaded from the MSigDB database (https://www.gsea-msigdb.org/gsea/msigdb). Gene set enrichment analysis (GSEA) was performed using the “clusterProfiler” package, and the top five most significantly enriched pathways were visualized using the “enrichplot” package.

### Statistical analysis

Statistical analyses were performed using parametric or non-parametric tests based on the distribution characteristics of the data. Normality was assessed using the Shapiro-Wilk test: data following a normal distribution were analyzed using independent samples t-tests and one-way analysis of variance (ANOVA), while non-normally distributed data were analyzed using the Mann-Whitney U test and KW test. P-values for multiple comparisons were adjusted using the Benjamini-Hochberg method to control the false discovery rate. Results are presented as mean ± standard deviation (for normally distributed data) or median with interquartile range (IQR) (for non-normally distributed data). LASSO regression was used to explore the significance of MMP8, DDX24, EMB, and RNASE2 in sepsis diagnosis. ROC curves were generated for binary classification prediction, and Spearman’s non-parametric test was employed to evaluate correlations between diagnostic genes and immune cell infiltration, as well as correlations among immune cells. An adjusted P-value of less than 0.05 was considered statistically significant and is indicated with asterisks (*padj < 0.05, **padj < 0.01, ***padj < 0.001, ****padj < 0.0001). Data analysis and visualization were performed using R software (version 4.3.3) and GraphPad Prism (version 10.6.1), and graphical editing was carried out using Adobe Illustrator.

## Data Availability

The datasets supporting the conclusions of this article are available in the National Genomics Data Center (NGDC) repository (accession number: HRA002988; https://ngdc.cncb.ac.cn/gsa-human/browse/HRA002988) and the Gene Expression Omnibus (GEO) repository (GSE65682: https://www.ncbi.nlm.nih.gov/geo/query/acc.cgi?acc=GSE65682; GSE57065: https://www.ncbi.nlm.nih.gov/geo/query/acc.cgi?acc=GSE57065; GSE185263: https://www.ncbi.nlm.nih.gov/geo/query/acc.cgi?acc=GSE185263; GSE134347: https://www.ncbi.nlm.nih.gov/geo/query/acc.cgi?acc=GSE134347; GSE69686: https://www.ncbi.nlm.nih.gov/geo/query/acc.cgi?acc=GSE69686). The codes used in this study were uploaded in github (https://github.com/bblc965/A-Transcriptome-Based-Modeling-Study.git), and available for download.

## Supplementary material

Supplementary Figure 1 GSE57065 calibration curve, DCA curve and CIC curve. **(A)** The calibration curve shows that the original curve and the corrected curve are relatively close to the 45°ideal line, indicating excellent model accuracy.**(B,C)** The DCA curve **(B)** and the CIC curve **(C)** show that when the threshold is within the range of 0.2 to 1, the model has clinical efficacy, and the corresponding cost-benefit ratio is 1:5 to 100:1.

Supplementary Figure 2 GSE185263 calibration curve, DCA curve and CIC curve. **(A)** The calibration curve shows that the original curve and the corrected curve are relatively close to the 45°ideal line, indicating excellent model accuracy. **(B,C)** The DCA curve **(B)** and the CIC curve **(C)** show that when the threshold is within the range of 0.4 to 1, the model has clinical efficacy, and the corresponding cost-benefit ratio is 3:4 to 100:1.

Supplementary Figure 3 GSE134347 calibration curve, DCA curve and CIC curve. **(A–C)** When identifying healthy controls and sepsis, the original curve and the corrected curve in the calibration curve graph are relatively close to the 45°ideal line, indicating good model accuracy **(A)**; the DCA curve and the CIC curve have clinical efficacy regardless of the threshold value, and the model performs well in this dataset **(B,C)**. **(D–F)** When identifying non-infection and sepsis, the original curve and the corrected curve in the calibration curve graph deviate from the 45°ideal line, indicating poor model accuracy **(D)**; the DCA curve and the CIC curve show that when the threshold value is within the range of 0.4-0.8, the model has clinical efficacy, and the corresponding cost-benefit ratio is 3:4 to 5:1**(E,F)**. **(G–I)** When identifying healthy controls and non-infection, the original curve and the corrected curve in the calibration curve graph are closely aligned with the 45°ideal line, indicating extremely high model accuracy; the DCA curve and the CIC curve show that when the threshold value is within the range of 0.2-1, the model has clinical efficacy, and the corresponding cost-benefit ratio is 1:5 to 100:1.

Supplementary Figure 4 GSE69686 calibration curve, DCA curve and CIC curve. **(A–C)** When identifying healthy controls and sepsis, the calibration curve shows that the original curve and the corrected curve deviate from the 45°ideal line, indicating poor model accuracy**(A)**; the DCA curve and the CIC curve show that when the threshold is within the range of 0.2-0.7, the model has clinical efficacy, and the corresponding cost-benefit ratio is 1:5 to 5:2**(B,C)**. **(D–F)**When identifying uninfected chorio and sepsis, the calibration curve shows that the original curve and the corrected curve deviate from the 45°ideal line, indicating poor model accuracy**(D)**; the DCA curve and the CIC curve show that when the threshold is within the range of 0.4-0.7, the model has clinical efficacy, and the corresponding cost-benefit ratio is 3:4 to 5:2**(E,F)**. **(G–I)** When identifying healthy controls and uninfected chorio, the calibration curve shows that the original curve and the corrected curve deviate from the 45°ideal line, indicating poor model accuracy**(G)**; the DCA curve and the CIC curve show that when the threshold is within the range of 0.3-0.4, the model has clinical efficacy, and the corresponding cost-benefit ratio is 2:5 to 3:4**(K,I)**. Considering the diagnostic efficacy AUC value of the model in the neonatal population, we believe that this model cannot be used for the diagnosis of neonatal sepsis, but only has a certain indicative effect.

Supplementary Figure 5 Immune infiltration landscape of sepsis by quanTIseq. **(A)** Diagram showing the proportions of 11 immune cell types using the transcriptomic dataset. **(B)** Bar graph showing the proportions of 11 immune cells in indicated group. **(C)** Bubble plot showing the correlation relationship of the 11 immune cell types. **(D–G)** Bubble plots showing the correlation relationship of MMP8, DDX24, EMB, and RNASE2 with immune cell infiltration.

Supplementary Table 1 Comprehensive validation of the diagnostic model: complete results across all metrics.

Supplementary Table 2 The test results and AUC values of individual biomarkers PGLYRP1, MMP9, CYP1B1, and BPI.

Supplementary Table 3 The diagnostic efficacy and accuracy of the PGLYRP1, MMP9, CYP1B1, and BPI diagnostic model.
