# Supplementary material for: Identification of MMP8, DDX24, RNASE2, and EMB as a novel diagnostic gene panel for sepsis: a transcriptome-based modeling study
Source: Front Immunol. 2026 Sep 7;17:1911134. doi: 10.3389/fimmu.2026.1911134 (PMC13590547; doi:10.3389/fimmu.2026.1911134)
Supplement: Supplementary file 1 [file SupplementaryFile1.pdf]

SUPPLEMENTARY FIGURE:

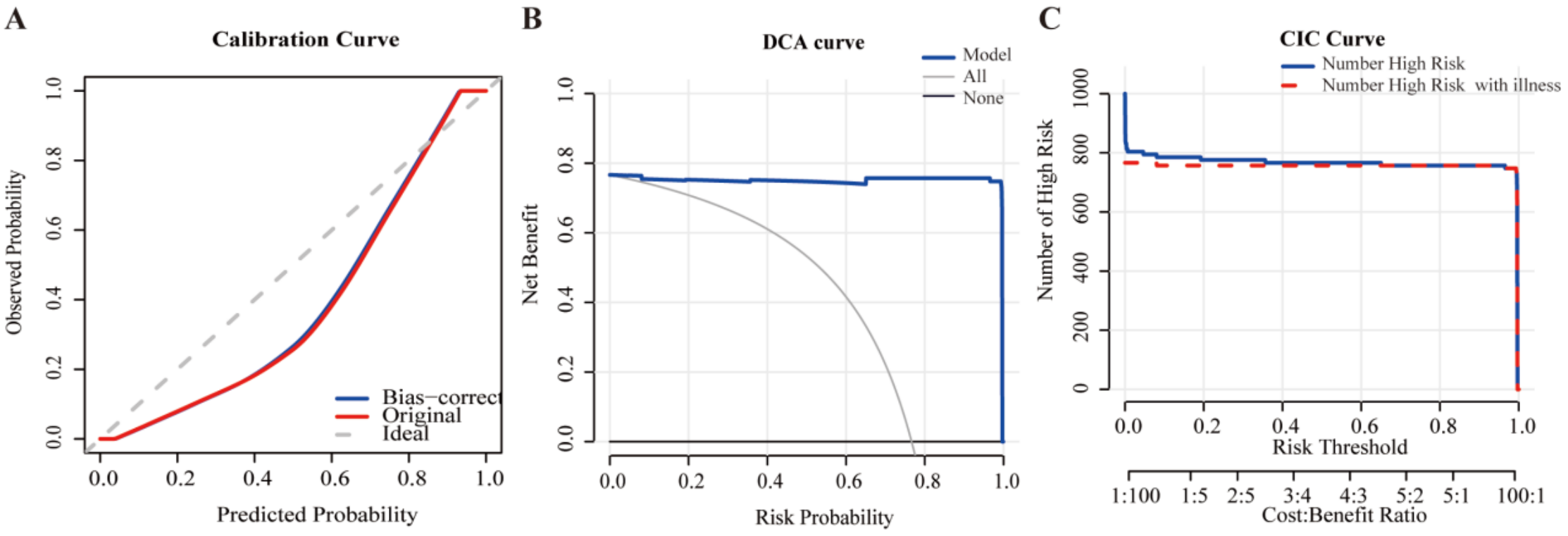

Supplementary Figure 1. GSE57065 calibration curve, DCA curve and CIC curve.(**A**)The calibration curve shows that the original curve and the corrected curve are relatively close to the 45° ideal line, indicating excellent model accuracy.(**B,C**) The DCA curve(B) and the CIC curve(C) show that when the threshold is within the range of 0.2 to 1, the model has clinical efficacy, and the corresponding cost-benefit ratio is 1:5 to 100:1.

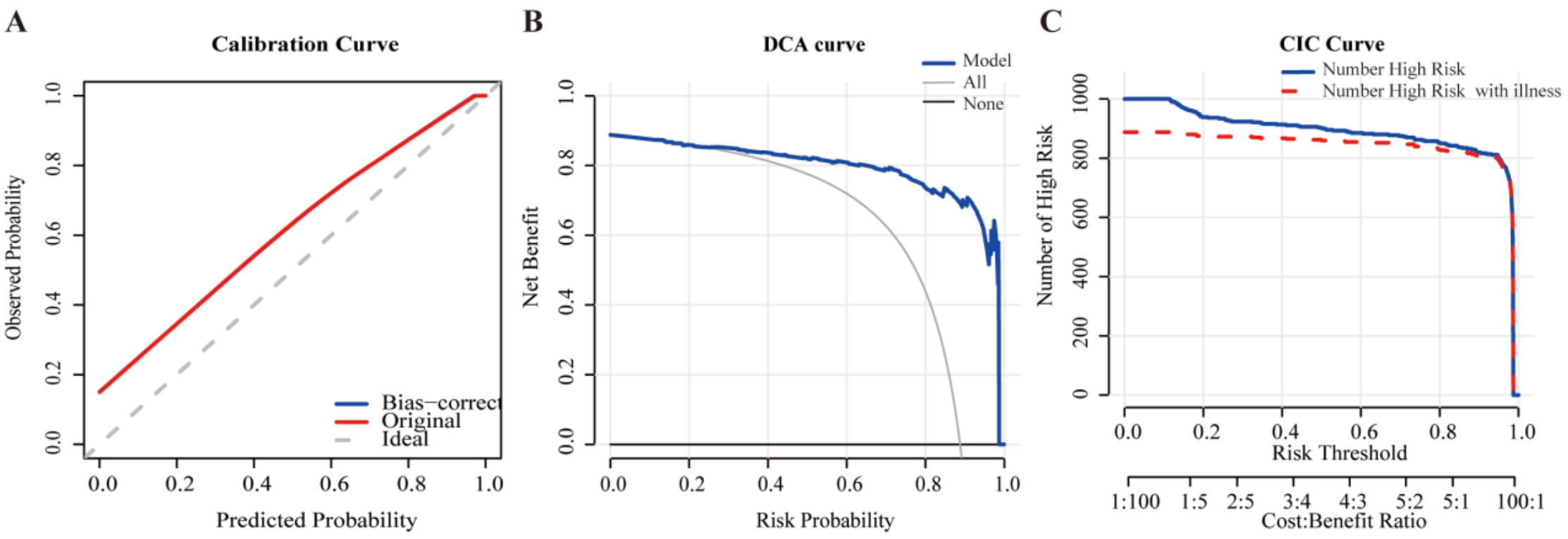

Supplementary Figure 2.GSE185263 calibration curve, DCA curve and CIC curve.(**A**)The calibration curve shows that the original curve and the corrected curve are relatively close to the 45° ideal line, indicating excellent model accuracy.(**B,C**) The DCA curve(B) and the CIC curve(C) show that when the threshold is within the

range of 0.4 to 1, the model has clinical efficacy, and the corresponding cost-benefit ratio is 3:4 to 100:1.

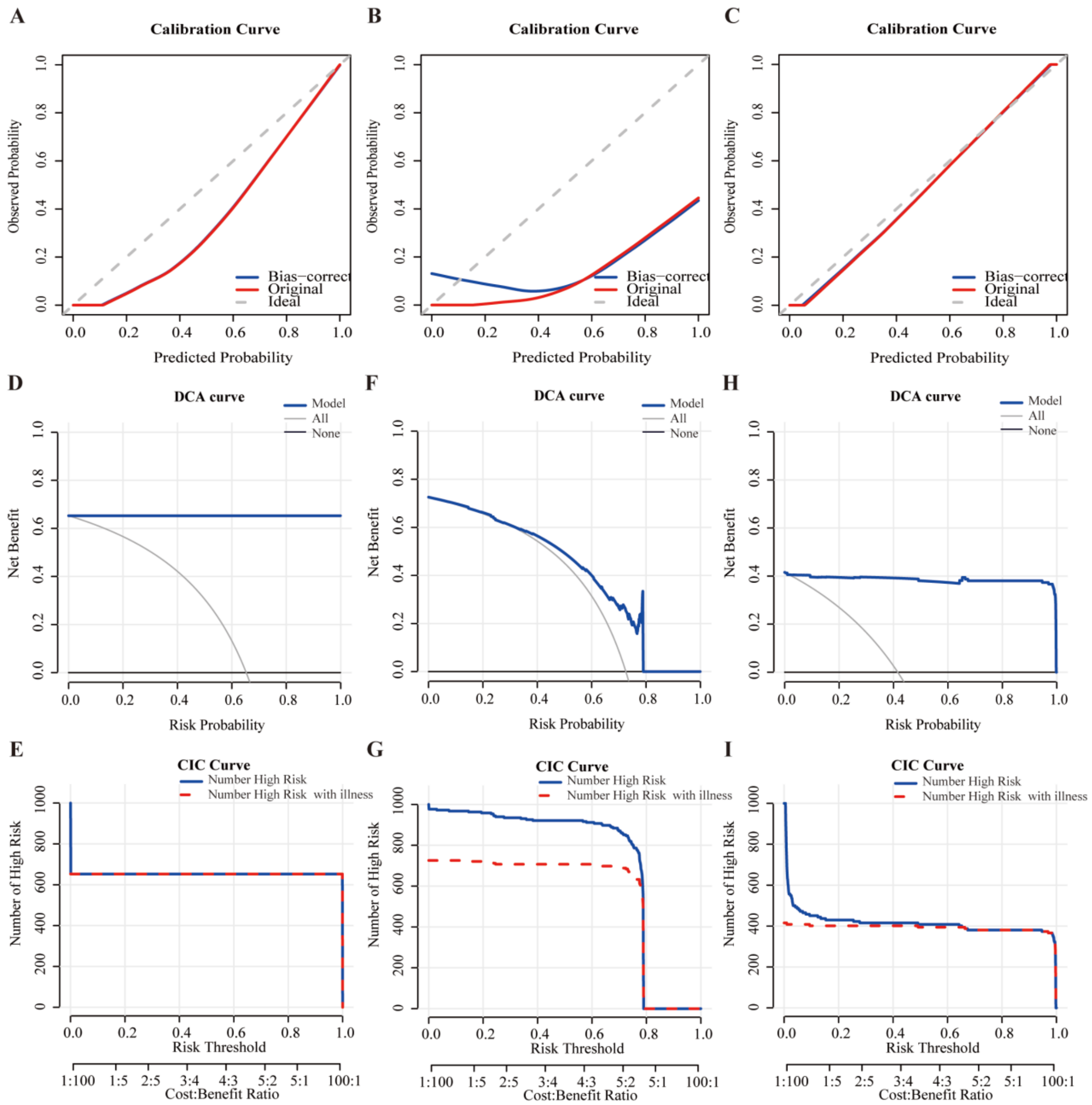

Supplementary Figure 3.GSE134347 calibration curve, DCA curve and CIC curve.(A-C) When identifying healthy controls and sepsis, the original curve and the corrected curve in the calibration curve graph are relatively close to the 45° ideal line, indicating good model accuracy(A); the DCA curve and the CIC curve have clinical efficacy regardless of the threshold value, and the model performs well in this

dataset(B,C). (**D-F**) When identifying non-infection and sepsis, the original curve and the corrected curve in the calibration curve graph deviate from the 45° ideal line, indicating poor model accuracy(D); the DCA curve and the CIC curve show that when the threshold value is within the range of 0.4-0.8, the model has clinical efficacy, and the corresponding cost-benefit ratio is 3:4 to 5:1(E,F). (**G-I**) When identifying healthy controls and non-infection, the original curve and the corrected curve in the calibration curve graph are closely aligned with the 45° ideal line, indicating extremely high model accuracy; the DCA curve and the CIC curve show that when the threshold value is within the range of 0.2-1, the model has clinical efficacy, and the corresponding cost-benefit ratio is 1:5 to 100:1.

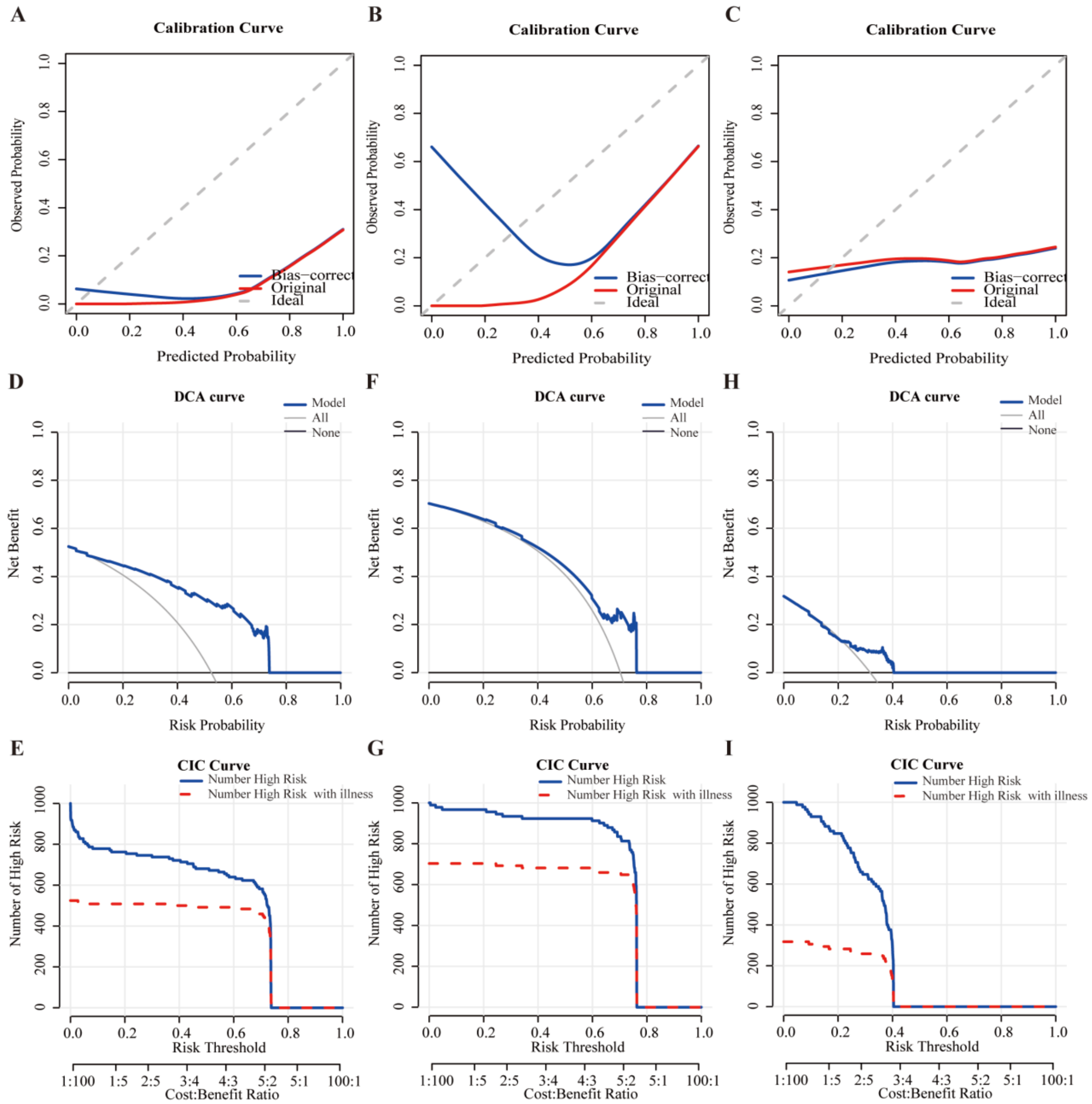

Supplementary Figure 4.GSE69686 calibration curve, DCA curve and CIC curve.(A-C) When identifying healthy controls and sepsis, the calibration curve shows that the original curve and the corrected curve deviate from the 45° ideal line, indicating poor model accuracy(A); the DCA curve and the CIC curve show that when the threshold is within the range of 0.2-0.7, the model has clinical efficacy, and the corresponding cost-benefit ratio is 1:5 to 5:2(B,C). (**D-F**)When identifying uninfected chorio and sepsis, the calibration curve shows that the original curve and the corrected curve deviate from the 45° ideal line, indicating poor model

accuracy(D); the DCA curve and the CIC curve show that when the threshold is within the range of 0.4-0.7, the model has clinical efficacy, and the corresponding cost-benefit ratio is 3:4 to 5:2(E,F). (**G-I**) When identifying healthy controls and uninfected chorio, the calibration curve shows that the original curve and the corrected curve deviate from the 45° ideal line, indicating poor model accuracy(G); the DCA curve and the CIC curve show that when the threshold is within the range of 0.3-0.4, the model has clinical efficacy, and the corresponding cost-benefit ratio is 2:5 to 3:4(K,I). Considering the diagnostic efficacy AUC value of the model in the neonatal population, we believe that this model cannot be used for the diagnosis of neonatal sepsis, but only has a certain indicative effect.

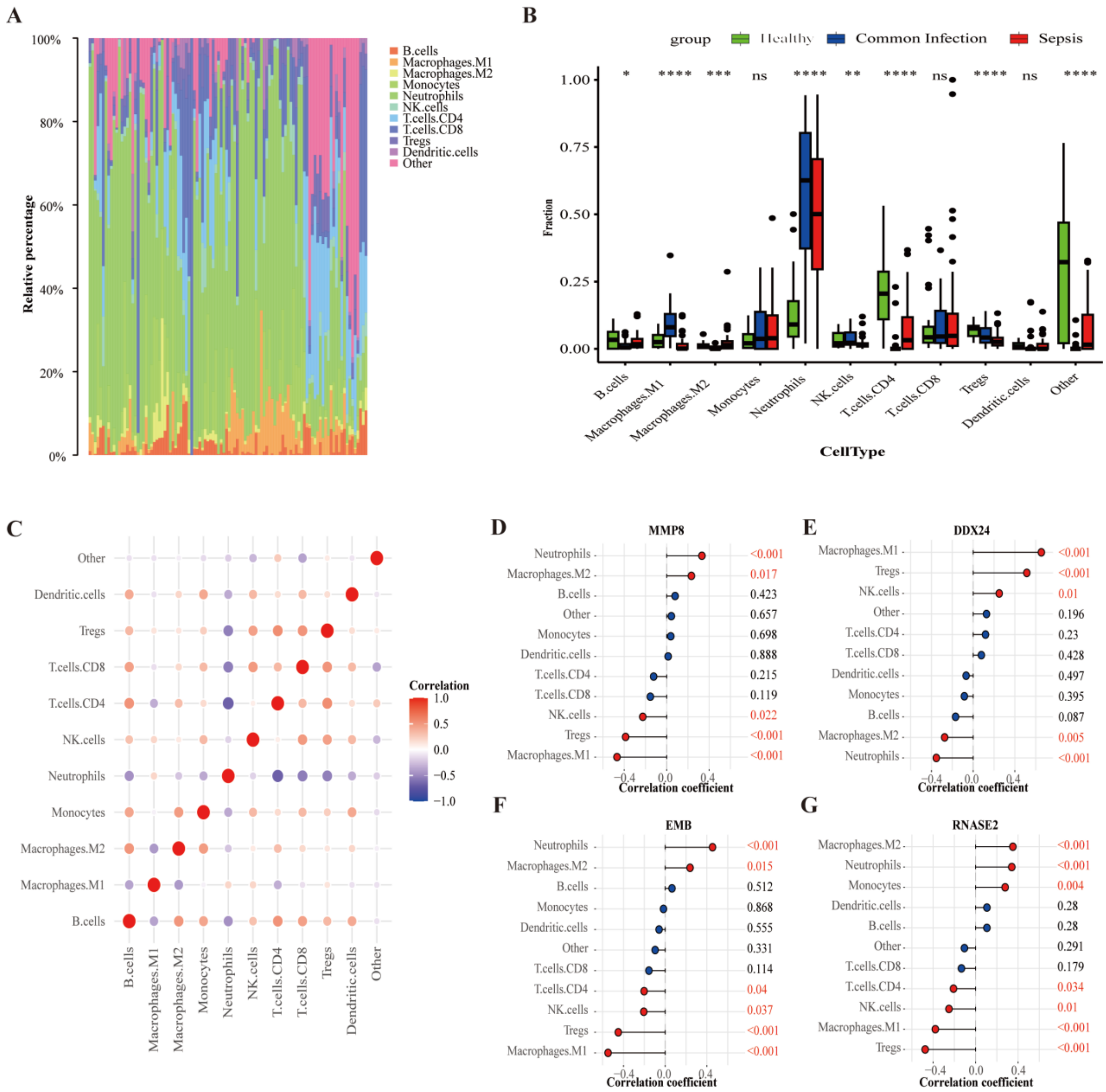

Supplementary Figure 5. Immune infiltration landscape of sepsis by quanTIseq. (A) Diagram showing the proportions of 11 immune cell types using the transcriptomic dataset. (B) Bar graph showing the proportions of 11 immune cells in indicated group. (C) Bubble plot showing the correlation relationship of the 11 immune cell types. (D-G) Bubble plots showing the correlation relationship of MMP8, DDX24, EMB, and RNASE2 with immune cell infiltration.

Supplementary Table 1. Comprehensive validation of the diagnostic model:

complete results across all metrics.

NPV	Cut-off	BSS	Model Brier Score	Null Model Brier Score	Calibration Intercept	Calibration Slope
0.672	2.167	0.803	0.01	0.05	-0.13	1.198
0.96	0.529	0.897	0.022	0.218	-0.427	1.743
0.964	0.774	0.754	0.057	0.231	-1.394	1.018
0.947	-4.293	-0.464	0.363	0.248	1.992	0.471
0.962	1.087	0.918	0.015	0.179	-1.265	1.721
0.556	0.837	0.48	0.052	0.1	0.786	0.879
1	0.852	0.964	0.008	0.227	-22.1	25.675
0.533	7.607	-0.209	0.241	0.199	-1.678	0.373
0.965	0.139	0.877	0.03	0.243	0.016	1.829
0.891	4.002	-0.393	0.347	0.249	-2.509	0.461
0.607	5.328	-0.239	0.258	0.209	-1.461	0.355
0.875	2.856	-1.34	0.507	0.217	-1.416	0.16

Dataset	Comparison	AUC	Sensitivity	Specificity	Accuracy	PPV
**GSE65682**	**Sepsis VS Health**	0.996	0.974	0.976	0.974	0.999
**PBMC RNA-seq**	**Sepsis VS Health**	0.997	0.98	1	0.987	1
	**Sepsis VS Common Infection**	0.986	0.98	0.931	0.963	0.962
	**Common Infection VS Health**	0.823	0.966	0.75	0.868	0.824
**GSE57065**	**Sepsis VS Health**	0.999	0.988	1	0.991	1
**GSE185263**	**Sepsis VS Health**	0.961	0.908	0.909	0.908	0.988
**GSE134347**	**Sepsis VS Health**	1	1	1	1	1
	**Sepsis VS Noninfection**	0.811	0.724	0.831	0.753	0.919
	**Noninfection VS Health**	0.989	0.949	1	0.979	1
**GSE69686**	**Sepsis VS Health**	0.852	0.922	0.707	0.82	0.776
	**Sepsis VS Uninfected Chorio**	0.769	0.828	0.63	0.769	0.841
	**Uninfected Chorio VS Health**	0.666	0.815	0.603	0.671	0.489

Supplementary Table 2. The test results and AUC values of individual biomarkers PGLYRP1, MMP9, CYP1B1, and BPI.

Dataset	Comparison	PGLYRP1	MMP9	CYP1B1	BPI
**PBMC RNA-scq**	**JT Test Padj**	＜2.23E-50	5.04E-12	5.73E-13	1.38E-09
	**Sepsis VS Health**	0.981	0.963	0.957	0.877
	**Sepsis VS Common Infection**	0.884	0.784	0.84	0.954
**GSE65682**	**Sepsis VS Health**	0.974	0.976	0.92	0.902
**GSE57065**	**Sepsis VS Health**	0.992	0.989	0.951	0.966

Supplementary Table 3.The diagnostic efficacy and accuracy of the PGLYRP1, MMP9, CYP1B1, and BPI diagnostic model

Model Brier Score	Null Model Brier Score	Calibration Intercept	Calibration Slope
0.018	0.05	-0.105	1.101
0.133	0.218	4.397	0.988
0.136	0.231	1.424	0.678
0.474	0.248	5.095	0.767
0.226	0.179	-17.954	2.607
0.44	0.1	3.379	0.42
0.335	0.227	-16.017	2.784
0.273	0.199	-2.42	0.406
0.564	0.243	-11.611	2.047
0.465	0.249	-4.595	0.563
0.296	0.209	-5.51	0.73
0.668	0.217	-1.918	0.157

AUC	Sensitivity	Specificity	Accuracy	PPV	NPV	Cut-off	BSS
0.988	0.959	0.976	0.96	0.999	0.569	2.092	0.625
0.992	0.922	1	0.947	1	0.857	-2.85	0.39
0.933	0.882	0.862	0.875	0.918	0.806	-1.813	0.413
0.905	0.966	0.75	0.868	0.824	0.947	-7.217	-0.914
0.999	0.988	1	0.991	1	0.962	7.592	-0.264
0.853	0.684	0.977	0.717	0.996	0.281	-2.545	-3.417
0.993	0.974	0.988	0.979	0.993	0.953	5.984	-0.478
0.685	0.699	0.559	0.66	0.807	0.412	8.093	-0.373
0.958	0.898	0.952	0.93	0.93	0.929	5.585	-1.321
0.802	0.891	0.724	0.811	0.781	0.857	7.963	-0.865
0.786	0.797	0.704	0.769	0.864	0.594	8.409	-0.418
0.641	0.778	0.569	0.635	0.457	0.846	6.571	-2.08

Dataset	Comparison
GSE65682	Sepsis VS Health
PBMC RNA-scq	Sepsis VS Health
	Sepsis VS Common Infection
	Common Infection VS Health
GSE57065	Sepsis VS Health
GSE185263	Sepsis VS Health
GSE134347	Sepsis VS Health
	Sepsis VS Noninfection
	Noninfection VS Health
GSE69686	Sepsis VS Health
	Sepsis VS Uninfected Chorio
	Uninfected Chorio VS Health
